# Which One? Choosing Favorite Robot After Different Styles of Storytelling and Robots’ Conversation

**DOI:** 10.3389/frobt.2021.700005

**Published:** 2021-09-09

**Authors:** Anna-Maria Velentza, Nikolaos Fachantidis, Sofia Pliasa

**Affiliations:** ^1^ School of Educational and Social Policies, University of Macedonia, Thessaloniki, Greece; ^2^ Laboratory of Informatics and Robotics in Education and Society (LIRES) Robotics Lab, University of Macedonia, Thessaloniki, Greece

**Keywords:** robot personality traits, expressive body movement, storytelling, conversation, human–robot interaction, robot characteristics, social robotics/HRI, knowledge acquisition

## Abstract

The influence of human-care service robots in human–robot interaction is becoming of great importance, because of the roles that the robots are taking in today’s and future society. Thus, we need to identify how humans can interact, collaborate, and learn from social robots more efficiently. Additionally, it is important to determine the robots’ modalities that can increase the humans’ perceived likeness and knowledge acquisition and enhance human–robot collaboration. The present study aims to identify the optimal social service robots’ modalities that enhance the human learning process and level of enjoyment from the interaction and even attract the humans’ attention to choosing a robot to collaborate with it. Our target group was college students, pre-service teachers. For this purpose, we designed two experiments, each one split in two parts. Both the experiments were between groups, and human participants had the chance to watch the Nao robot performing a storytelling exercise about the history of robots in a museum-educational activity *via* video annotations. The robot’s modalities were manipulated on its body movements (expressive arm and head gestures) while performing the storytelling, friendly attitude expressions and storytelling, and personality traits. After the robot’s storytelling, participants filled out a knowledge acquisition questionnaire and a self-reported enjoyment level questionnaire. In the second part, we introduce the idea of participants witnessing a conversation between the robots with the different modalities, and they were asked to choose the robot with which they want to collaborate in a similar activity. Results indicated that participants prefer to collaborate with robots with a cheerful personality and expressive body movements. Especially when they were asked to choose between two robots that were cheerful and had expressive body movements, they preferred the one which originally told them the story. Moreover, participants did not prefer to collaborate with a robot with an extremely friendly attitude and storytelling style.

## Introduction

Social robots are employed in a variety of roles that require social skills, such as teaching, guiding tours, and general interaction with humans. The robot’s ability for verbal and nonverbal communication, expressed through different modalities, is key to successful human–robot interaction (Bartneck et al., 2020). The substrate and the influence of the embodiment of social robots affect humans’ attitudes toward them and their decision making. Furthermore, humans who interact with physically embodied robots perceived significantly higher trust, attachment, and credibility from the robots’ behavior (Wang and Rau, 2019; Dziergwa et al., 2018). Facial expressions are the most well-studied emotional expression. Humanoid robots who cannot imitate facial expressions can still influence decision making and form cooperative relationships with humans by using speech, colors, and full-body motion (Takahashi et al., 2021). Expressive body language (Marmpena et al., 2018; Zhang et al., 2018) influences humans’ arousal, their attitude toward robots, and perceptions from their interaction. The robot’s speech is another important factor, both auditory and semantic, that is used for expressing emotional behavior in the human–robot interaction (Valenti et al., 2020). However, it is not clear which modality (personality, body language, or speech) is more effective when a social robot performs an educational storytelling activity.

This study aims to bridge this gap in the literature. Participants were exposed to a video storytelling of an individual Nao robot featured with specific personality characteristics, and after testing their gained knowledge and level of enjoyment from its storytelling, they witness a conversation between the same robot and other Nao robots featured with different personality characteristics. After the conversation, they chose the preferred one, which might be the same Nao robot or another one featured with different modalities (i.e., friendly speech style or intense body movements). We introduce to the participants four different Nao robots, featured with different modalities, which are as follows: a) serious personality, b) cheerful personality, c) cheerful personality with enriched intense body movements, and d) cheerful personality enriched with extremely friendly storytelling. The quality aspect evaluates how enjoyable the storytelling was for the participants and is, most importantly, the criterion that defines the selection of the Nao robot. This study differs from previous human–robot interaction studies, as it combines the following: (a) the evaluation of robots with different modalities in terms of the participants’ knowledge acquisition and level of enjoyment from their storytelling, (b) the evaluation of robots after having a conversation with similar robots exhibiting different modalities/features, (c) self-reported intention of future collaboration with a robot based on its modalities, and, most importantly, (d) a broad range of modulated robot features.

In our first experiment, we used video annotation showing the Nao robot performing educational storytelling on the history of specific robots in front of a museum environment, by expressing either a serious or a cheerful personality. The serious personality follows the norms of Ullrich (2017) and Robert et al. (2020), where a serious robot expresses a professional/efficient character. Following the same authors’ recommendations, the cheerful robot mimics the behavior of an enthusiastic, extroverted person. The robot’s personality was manipulated through different body languages, voices, and storytelling styles. The experimental environment is a museum/art gallery used for educational purposes. We make the common assumption that the purpose of storytelling in a museum is to pleasantly convey information about the exhibits. Thus, we stress the two personality robots based on the participants’ scores in the following: (a) a knowledge acquisition test and (b) the level of enjoyment based on a Likert scale questionnaire. Several studies investigated differences between robots’ personalities and found that they should be in line with the performed activity. Goetz suggested the use of robots with a cheerful personality for human–robot collaboration in joyful activities and robots with a serious personality for serious business-oriented tasks (Goetz, Kiesler, and Powers 2003). The need for serious, assertive personality traits was confirmed by participants’ intention to collaborate with an AI interviewer with a corresponding personality for a high-stakes job interview (Zhou et al., 2019).

Second, after separately evaluating each robot, participants watch a video with two robots, one serious and one cheerful, discussing their work, personality traits, modalities, and a short storytelling performance about the history of a specific robot. The conversation aims to give the participants the chance to compare the different robots’ characteristics and have a clear view regarding the personality manipulations. We ask the participants to choose their preferred robot to continue the storytelling. In previous studies, researchers implemented collaborative robot tasks, such as stand-up comedy (Hayashi et al., 2008) or collaborative tour guide robots (Iio, et al., 2017; Velentza et al., 2019; Velentza et al., 2020), as well as comparison of different modalities in android robots (Mikata et al., 2019). Dialogue systems consisting of two robots can enhance elderly people’s attention by giving the chance to hold longer conversations in care homes (Nishio et al., 2021). In our experiment, we introduce the novel idea of having a conversation with three robots talking about their selves and abilities to make participants know them better. Studies with real-life university lectures performed by the Nao robot showed that participants had significantly different opinions about the ideal robot characteristics before and after listening to a lecture (Velentza et al., 2020). Based on those findings, the participants must witness the robots in action before choosing the preferred one. Furthermore, we investigated whether participants’ previous experience with one robot (either the cheerful or the serious one) made them biased in favor of it and asked them to complete a multiple-choice and open-answer questionnaire regarding the robot’s modality that led them to this choice.

The most important findings of Experiment I, the comparison between a cheerful robot and a serious robot, lead us to design and implement Experiment II by deconstructing the modalities of the cheerful robot that was the clear winner with regard to the participants’ preference. The modalities that made the cheerful robot preferable were as follows: (a) expressive body movements and (b) friendly storytelling. Based on these findings, we replicated the same experiment, but this time, we concentrated on three cheerful robots with different uplifting modalities. The first robot had intense expressive body movements, the second one performed in a very friendly manner with extremely friendly storytelling, and the third one was the cheerful robot that participated in Experiment I. This experiment therefore investigates the modality that is preferable for the human participants. The results of this study will help robot designers, constructors, and researchers to emphasize, when possible, the more effective modalities to achieve an efficient human–robot interaction.

The machines’ and robots’ personality has personality traits attributed from the human personality. However, the ways and theories used to design personality into robotics vary widely. Researchers from multiple academic disciplines have tried to assign personality traits to social robots by implementing vocal, behavioral, linguistic, or visual perspectives. A recent meta-analysis suggested that robots have four types of personality; extraversion thinking and/or feeling and introversion thinking and/or feeling (Mou et al., 2020). However, robotics researchers, borrowing ideas from the field of psychology, proposed and applied technology-oriented models, such as the co-determination of the robot’s emotions based on their performed activity, certainty, and perceived pleasantness (Mou et al., 2020). Specific traits have been associated with a robot’s personality, such as being friendly, dominant, aggressive, or shy (Hiah et al., 2013). In other studies, personality was simplified into three modes: positive, negative, and neutral. Language is a tool to fit those profiles. To express a positive personality, the robot can be friendly, show enthusiasm about everything, be nice, and compliment humans, while for expressing a neutral personality, the robot acts like a machine/robot/computer in a stereotypical sense by focusing on efficiency (Ullrich, 2017). Those robot modes have also been found in the HRI studies as cheerful (playful or joyful) by expressing friendly, enthusiastic, and extroverted human traits or serious, highlighting the robot’s efficiency and professionalism (Robert et al., 2020).

A robot’s personality can be expressed by verbal and nonverbal behavior. The verbal behaviors focus on the robot’s voice and storytelling style, while the nonverbal behaviors are more complex—involving body movement, facial expressions, gestures, and posture. Saunderson and Nejat (2019) summarized the robot’s nonverbal behavior into the term “kinesics.” Kinesics encloses a highly informative capacity, which along with verbal communication can express emotional states and interpersonal and social behaviors. Arm gestures are important for practical purposes, that is, pointing out, emphasizing, and highlighting (Cienki and Müller, 2008) but also in expressing feelings, especially in a storytelling procedure (Striepe et al., 2019). Robots tend to receive more positive feedback when they accompany their storytelling with arm gestures, even though these gestures can be completely irrelevant to the context of their speech (Salem et al., 2012). Regardless of congruency or incongruency between gestures and the storytelling context, they contribute to the robot’s perceived humanlikeness, likability, and, more importantly, humans’ willingness to interact with the robot in the future (Aly and Tapus, 2016). The Nao robot, after a 10-min lecture about robots expressing intense body movements, is perceived as warm and capable (Peters et al., 2017). Moreover, robot arm gestures increase humans’ recall from the storytelling. Participants were able to recall approximately 10% more specific information when they experienced storytelling with a robot doing indicative gestures relevant to the story (van Dijk, Torta, and Cuijpers 2013). Apart from arm gestures, whole-body movements, such as dancing or nodding, together with the robot’s storytelling, increase the perceived robot’s anthropomorphism, likeability, and intelligence, in comparison to non-movement conditions (Rosenthal-von der et al., 2018).

Mikata et al. (2019) investigated the personality of individuality between two social robots by manipulating their appearance, voice, and behavior and evaluated how each modality affected the observers’ impression of the robot. Results showed that hand motion modality was a crucial factor for expressing a robot’s personality. Most importantly, robots that expressed themselves with a combination of modalities were perceived as more likable. Löffler et al. (2018) developed a simple robot to explore the influence of different modalities in users’ acceptance, including variations of postures, gestures, head movements, and emotion representations (Löffler, Schmidt, and Tscharn 2018). Although the emotional expression of a robot plays a significant role in how it is perceived, researchers also highlighted the importance of the context, as social robots are expected to act according to the social expectations (Fischer et al., 2019). The effect of a social robot’s cues and body language expressions for having an affective human–robot interaction was also investigated in terms of the robot’s ability to mimic human behavior (Stoeva and Gelautz, 2020). In our study, we will evaluate the robot’s movements, not only for its ability to mimic human behavior (Stoeva and Gelautz, 2020), and we will go further and evaluate the participants’ acceptance (Löffler et al., 2018) or impression (Mikata et al., 2019).

A social robot shows empathy, reasoning, and emotion mainly using dialogue. Humanlike dialogue is crucial for robots that engage in social roles, as we expect them to show humanlike behaviors (Kawahara, 2019). Children–robot interaction studies reported that during educational activities, children self-reported a similar level of learning and liking from an expressive and a flat storytelling robot. However, the children’s facial expressions revealed a higher level of concentration and engagement during the robot’s expressive storytelling (Westlund et al., 2017). A robot’s storytelling ability helps them serve as educational tools and can motivate disabled children to achieve their therapeutic goals (Chen et al., 2011). Moreover, the robot’s storytelling style determines the perceived acceptance and psychological anthropomorphism of the robot (Eyssel et al., 2012).

We are going to incorporate different body movements (expressive and intensely expressive) in a cheerful robot and evaluate the participants’ gained knowledge, level of enjoyment, and intention to collaborate with the robot again in the future. They will have the ability to assess the robot’s characteristics not only based on individual storytelling but also from a conversation between three robots expressing different characteristics. Moreover, we will integrate the robot’s storytelling style as an educational tool as proposed by Chen et al. (2011), and we will stress three different storytelling styles: serious-professional, cheerful-friendly, and cheerful-extremely friendly. Additionally, apart from the perceived acceptance (Eyssel et al., 2012), we will evaluate the participants’ intention to see the robot again in the future after witnessing a conversation between robots with two or three different storytelling styles.

### Research Focus and Hypothesis

The main experimental questions investigated in the current study are as follows: (a) whether a short-term interaction with a social robot can build familiarity and lead participants to choose the robot they “knew” and met initially, in comparison with other robots, and (b) whether robot personality traits, modalities and expressions, such as movements and storytelling style, influence the participants’ choice when witnessing a conversation between the robots with different modalities. Previous studies showed that humans create emotional attachment toward a social robot during short-term quality activities (Robert 2018). This leads to hypothesis 1:

H1. Participants will decide to continue the storytelling activity with the robot they first experienced the storytelling with.

Previous studies showed that humans prefer to collaborate with a cheerful-personality robot, especially for cheerful activities (Goetz et al., 2003; Velentza et al., 2019; Velentza et al., 2020). This leads to hypothesis 2:

H2. We expect that in the case where H1 is not confirmed, most participants will choose the cheerful-personality robot to continue their activity.

In previous studies, researchers evaluated the effect of the robot’s voice, humanlike or machine-like, its embodiment, or the participants’ gender and personality type on participants’ learning outcome from the robot’s storytelling (Cruz-Maya and Tapus 2016) or their scores in lexical entertainment tasks based on the participants’ five big personality traits (Brandstetter et al., 2017). In our case, we are going to examine the participants’ learning outcome based on the robot’s expressed personality type (cheerful or serious) or modality (expressive body movements and extremely friendly storytelling). Task performance is influenced by the participants’ attention span (Lindsay and Miller, 2018). Moreover, relevant studies from the first author of this study (Velentza et al., 2019; Velentza et al., 2020) showed that participants in a memory test after a guiding tour had higher memory scores when the robot tour guide expressed a cheerful personality. Therefore, we believe that participants will have higher knowledge acquisition scores in the robot conditions under which they will later prefer the robot to continue with the storytelling. Therefore, hypothesis 3 is as follows:

H3. We expect that participants acquire more knowledge from the most preferable robot conditions.

The enjoyment of the learning procedure is strongly correlated with the choice of students following enjoyable courses in their future careers (Wang et al., 2021). Moreover, bringing out a positive mood increased the students’ evaluation of their teachers (Fortunato and Mincy 2003). Based on this, we expect participants to evaluate more positively and thus assign larger scores in the enjoyment level questionnaire to the cheerful or perceived-as-more-entertaining robot conditions in comparison with the serious ones, resulting in our fourth hypothesis:

H4. Participants will have greater enjoyment level scores under the cheerful robot conditions.

Humans develop an emotional connection with robots that move during their interaction (Lambert et al., 2019). Moreover, humans report higher perceived physical presence, helpfulness, emotion, and positive attitude toward a robot with limited expressivity added to its virtual arms (Groechel et al., 2019). A friendly attitude is also important as in the human–machine interaction, a personalized experience with an intelligent device makes people feel more comfortable when they perceive the device/machine as trustworthy (Karat, Bloom, and Karat 2004). The human–robot interaction with a robot with a socially friendly attitude in a science museum for 9 min made 95% of visitors express a desire to see the robot again in the future (Iio et al., 2020). Therefore, the fifth hypothesis is as follows:

H5. We must investigate which modality, expressive movements, or friendly storytelling will outperform the other in terms of the participants’ preference, knowledge acquisition, and level of enjoyment. Our alternatives are as follows: a) the expressive-movements robot condition will outperform the friendly-storytelling robot condition, b) the friendly-storytelling robot condition will outperform the expressive-movements robot condition, and c) the robots will have equal results.

## Materials and Methods

### General Method

#### Sampling Method

**FIGURE 1:**
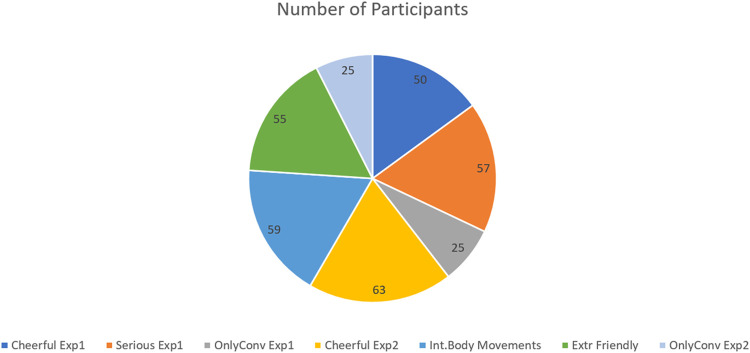
Total number of participants per condition.

The participants were college students (pre-service teachers) from the first to the last year. Their age and gender are representative of educational studies in Greek universities, where the experiments took place. Participants were recruited from the students who enrolled in the courses “Basic Principles of ICoT I and III” (compulsory courses). To avoid the “novelty effect,” where people unfamiliar with the robots have different reactions in human–robot activities due to the lack of experience in comparison with long-term interactions (Bartneck et al., 2020), all students who participated in the study had previously experienced a lecture with the Nao robot through previous studies of the authors. The students had to be registered into the courses to receive the link with the experiment, and we asked permission from the students for this experiment in advance. They were told that the experiment was a voluntary course exercise without a marking scheme and without bonus credits. They were all randomly assigned to each condition. The researchers applied an Excel-based pool technique, choosing randomly from a pool with their student ID number. The total number of participants was 225. More information will be given before every experimental design. In Figure 1, a graphical representation of the total number of students participating in each condition can be found.

#### Stimuli

We used the humanoid Aldebaran Nao robot for the experiments. Each robot was named after a Greek alphabet letter, aiming to avoid any identification that could bias the participants regarding the robot’s personality traits or modalities. The Nao robot has been successfully employed in a variety of social human–robot interaction applications, such as teaching in university classrooms (Xu et al., 2014) to Shakespearean theatrical performances with older adults (Greer et al., 2019). The robots’ movements and voice were programmed *via* Python scripts in the Choreographer 2.1.4 environment. To compare the robots’ personality traits, we manipulated their body language and storytelling style. The Nao robot cannot show facial expressions, and previous experiments (Xu et al., 2014) showed that students exposed to Nao robots with different personalities fail to recognize the differences between positive and negative moods. Nevertheless, the self-reported level of arousal and valence depended on the robots they interacted with (Xu et al., 2014). To program the robot’s movements, we used similar arm gestures to those used by (Xu et al., 2015) and body movements designed to express happiness in the Nao robot (English, Coates, and Howard 2017). In order to examine the appropriateness of the developed robot programs, the researchers shared the video of the individual robot’s storytelling with a group of ten academic staff members and ten students and asked them to describe the robot’s personality, without giving them any clue relevant to it. They all successfully recognized the personality of the robot when they were asked to watch the videos and try to describe it. Similarly, the three cheerful robots managed to give the appropriate characteristics to the robots. They were asked to give a name to each robot based on its behavior. They gave to the extremely friendly storytelling robot names such as “pal, my friend, dude,” to the intense moving robot, “the moving, the dancer, the high energy,” and for the cheerful, they used similar descriptions to those used for the comparison between the cheerful and the serious. Additionally, we designed a Likert scale questionnaire, asking the participants to evaluate the robot’s personality after watching a short video (3 min from the experimental design, the robot doing storytelling for two robots). The evaluation was between-groups. There, we included synonym words describing the personality based on the work of Robert et al. (2020) such as “cheerful,” “joyful,” “serious,” and “business. We separated the words which describe a robot as cheerful and those which describe a robot as serious, and we analyzed the participants’ answers with a *t*-test. The test indicated that the participants successfully recognized the cheerful robot with the cheerful words in comparison with the serious robot (t_(46)_ = −25.9, *p* = < 0.001, d = 11.09). The case was similar for the serious description in favor of the serious robot in comparison with the cheerful robot (t_(46)_ = 33.52, *p* = < 0.001, d = 11.11).

**FIGURE 2:**
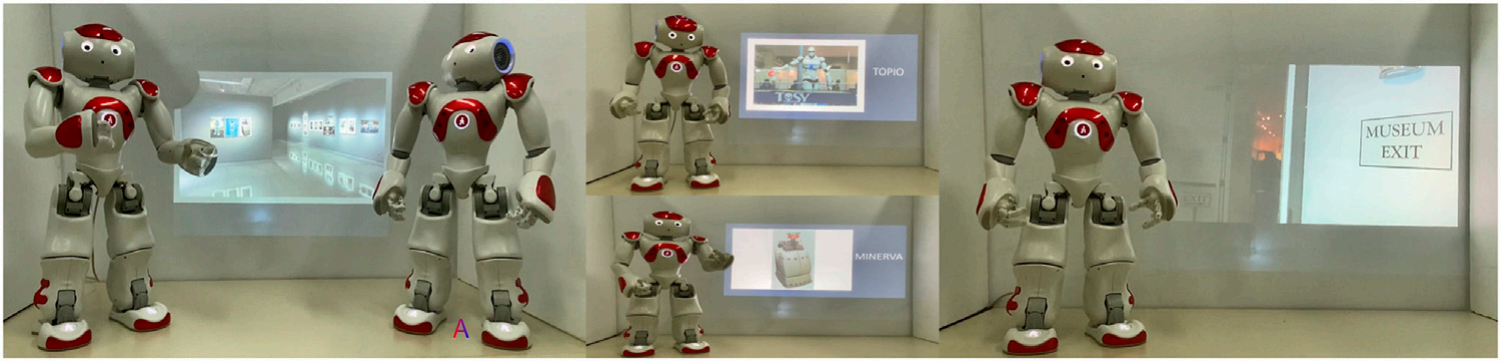
Exhibition context.

The robots performed their storytelling in a custom stage environment, mimicking a virtual museum design, following the recommendations of Patel et al. (2003). We presented images from a PowerPoint presentation with the aid of an Epson high-resolution projector, showing the following: (a) each exhibit, separately, while the robot was presenting it, (b) the inside, and (c) the outside of the museum, as shown in Figure 2.

The robot(s)’ performance was filmed using a high-resolution DSLR camera, and Wondershare Filmora X software^1^ was used for video editing. For the videos where the robots were in conversation with each other, we included the name of the robot in a subtitle each time they were talking, as depicted in the left image in Figure 1 and in the accompanying video. The videos of the robots were sent *via* email and evaluated by ten independent professionals, all of whom confirmed that each robot clearly reflects its personality. Consequently, different types of robot personalities could be recognized during robot-conversation sessions.

The robots’ voices were generated using the default machinery Nao voice with slight differences in speed and voice-shaping parameters, following the social identity theory in human–robot interactions (Edwards et al., 2019). The first experiment presented a comparison between the cheerful and the serious personalities, which were reflected in the robots’ voices (Nass and Lee, 2000) by asserting the same speed and different voice shaping. The serious robot had a voice with 20% more depth, based on voice personality stereotypes (Metze, Black, and Polzehl 2011). In the second experiment, where all robots had a cheerful personality, the voice shaping and speed varied by 2–5% for each robot, so as to have an almost unnoticeable difference among the robots’ voices, especially during the robot-conversation session (Polzehl, Moller, and Metze 2011).

#### Storytelling

The term “storytelling” describes the activity of sharing a story about events or objects, which can be slightly enriched and/or exaggerated. In our case, storytelling refers to the talk about robots given by the Nao robot, and it incorporates different characteristics in each condition. The content of each robot’s storytelling refers to eight different robot stories that appear sequentially, projected onto the white wall behind the robot (Figure 2). The storytelling was sequentially performed in this order for the following robots: Topio, Asimo, General Atomics Predator, Big Dog, Icub, Robonaut, Minerva, and Nao. Information was provided regarding their constructors, history, functionality, purpose, hardware and software characteristics, and fun facts. The content was fully understandable by non-experts. Each story lasted for approximately 10 min and involved critical information about each robot. The differences in the stories and thus our experimental manipulations were that the robots were following the cheerful script that made them show excitement in their speech regarding the presented robots, using phrases such as “this is amazing,” “I am so happy to present…,” etc., and also making jokes and personal comments. This gave the impression that they were enjoying their job and the interaction with the participants. The total number of sentences in the storytelling script was 84. In the cheerful script, 39/84 include a cheerful phrase (f = 0.464). More specifically, the cheerful robot from the “extremely friendly storytelling” condition did all the above by frequently adding phrases such as “friend,” “pal,” and “my friend,” addressed to the participants. In the cheerful robot script, the word “friend/pal” appeared in eight out of the 84 sentences (f = 0.095), while in the extremely friendly robot script, it appeared in 35 out of 84 (f = 0.416). In the serious script, the robot was professional, without expressing any positive (nor negative) feeling, enthusiasm, or personal comments. Under the cheerful conditions, the storytelling lasted 2 min longer because of the additional phrases mentioned by the robot. An extended example of the script is provided in *Storytelling Example and Storytelling Example*.

The individual storytelling of each robot was followed by a conversation between them regarding their experience with the participants, explaining their storytelling style and giving a demonstration of their different techniques. Those who participated in the first experiment and experienced the storytelling of the cheerful or the serious robot had the chance to watch 4.3 min of conversation between those two. In contrast, those who participated in the second experiment, in any of the three cheerful robot conditions, had the chance to witness a 5.4-min conversation between the three of them. This is important for the participants to have a clear idea about each robot’s unique characteristics in terms of personality or movement behavior to pick one of them to continue the storytelling. Examples of the conversations are provided in *Storytelling Example, Storytelling Example*, and accompanying videos.

#### Procedure

**FIGURE 3:**
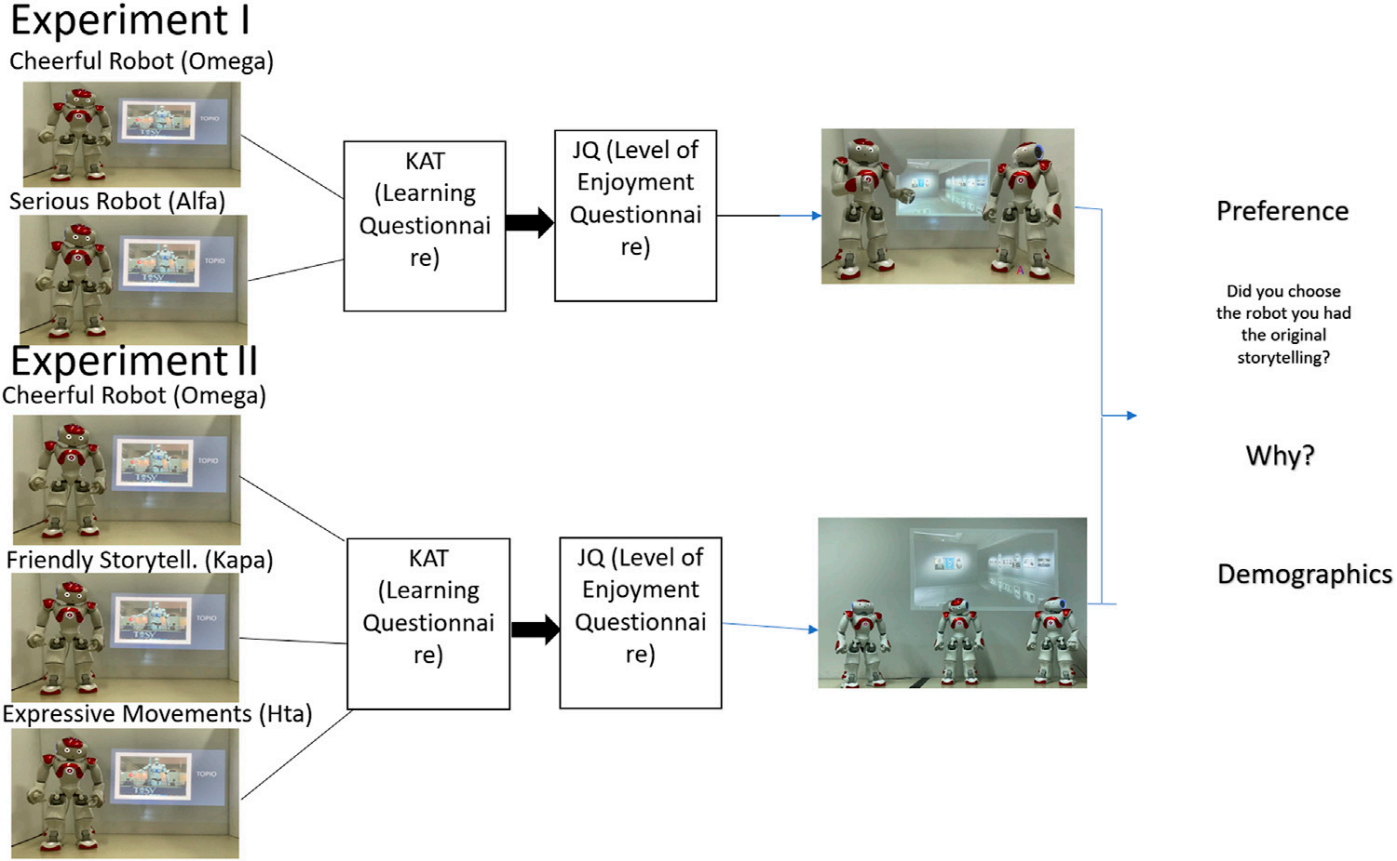
Procedure representation.

Following the recommendations of Bartneck et al. (2020), we first defined the context of the interaction as an educational storytelling museum guide. Starting with the technical details, a video annotation of one robot with different modalities was sent to the academic email address of the enrolled students *via* Google Drive sheets. The students who were randomly assigned to participate in Experiment I received a video with either the cheerful- or the serious-personality robot. They were able to watch the video only once, and the link was automatically deactivated after the first opening. A button that appeared at the bottom of the page led them to the first questionnaire, testing their knowledge acquisition, and after submitting it, it led them to the enjoyment level questionnaire. All the questions were compulsory, and when they were all answered, the video with the two robots’ conversation appeared on the screen. Participants were instructed by the robots at the end of the video to choose one of them to recount another story. Finally, the participants filled in the demographic and preference questions (2.1.4.3) and submitted the form. A graphical example of the procedure is shown in Figure 3. Similarly, the students who participated in Experiment II received one of the three video annotations: a) intense expressive body movements, b) extremely friendly storytelling, or c) cheerfulness from Experiment I, which served as the controlled condition. Finally, they watched a video with the three robots having a conversation. There were two additional groups of participants, who watched only the videos where the robots talked to each other, which served as a control condition for H1. The professor that taught the courses where the students were enrolled explained the procedure, and detailed descriptions were written at the beginning of each section of the Google sheet. Participation was voluntary and anonymous, and participants were not in danger of any harm. Regarding the robot(s)’ storytelling, the video started with the robot introducing itself and explaining the procedure. After they completed the storytelling, the robots said goodbye to the participants, promising that they were going to see them again later. The experimental design and procedure received approval from the ethics committee of the University of Macedonia.

#### Design

Experiment I had three conditions: a) a cheerful robot, b) a serious robot, and c) control, where participants chose a robot only after seeing the robots’ conversation without any prior knowledge of the task. In Experiment II, there were four conditions: a) a cheerful robot, b) intense body movements, c) extremely friendly storytelling, and d) control. In the control condition, participants were asked to choose the robot that they consider more appropriate for an educational guiding tour task. In all conditions of both Experiments I and II, different people participated (between participants). The robots performed the activities and were filmed in the Laboratory of Informatics and Robotics in Education and Society (LIRES). The experiments were designed for physical interaction between a participant and a robot in a laboratory environment. Unfortunately, due to Covid-19 restrictions, participants were not allowed to be on the university campus. Thus, appropriate modifications were made to deliver the videos. The evaluation of robot agents was successfully conducted *via* video annotations in other studies (Walters et al., 2011; Li et al., 2016).

##### Knowledge Acquisition Test

The knowledge acquisition test (KAT) examined the gained knowledge of the participants for specific information on six out of eight robots, excluding the first (Topio robot) and the final (Nao robot) to eliminate the serial position effect (Oberauer, 2003). Based on the serial position effect, participants can recall more information from the first and last items of a list or storytelling sequence. The KAT had 30 multiple-choice questions, with four potential answers, out of which only one was correct. We included 30 questions in the KAT questionnaire after performing a pilot study where we posed 56 questions to 40 people. Following the approach taken by Walker (1997), who listed criteria for choosing the more appropriate methodology of a study, we decided to exclude questions that had a response rate of <30 and >80%. After the participants watched the storytelling and proceeded to the KAT, the questions were grouped based on the robot they referred to, listed under a heading with the robot’s name. The first group of questions concerned the Asimo robot, which was the second in the storytelling sequence. The final questions were about the Minerva robot, which was the seventh in the row. Based on color studies, the background color of a questionnaire can affect participants’ memory and thus, following the recommendations of Dzulkifli (Dzulkifli and Mustafar, 2013), the KAT graphical interface was designed with color shades that enhance the participants’ attention without affecting their memory.

##### Level of Enjoyment

To evaluate the participants’ level of enjoyment from the robot’s storytelling, we used the Aesthetic Valence Questionnaire given by Velentza et al. (2020), which was used to measure the level of enjoyment of participants’ experience from a tour guide robot performance and storytelling about museum exhibits. The questionnaire has 35 questions which appeared all together on the screen, and participants evaluated their experience on a Likert scale from 1-Strongly Disagree to 5-Strongly Agree, described by single words such as “interesting,” “inspirational,” and “disgusting.” They were able to choose only one statement, and they were instructed to choose the one closer to what they felt during the storytelling.

##### Demographic and Final Choice Questions

After participants watched the final video with the robots’ conversation, we asked them to perform the following tasks:

1) Choose one of the robots to continue the storytelling. We provided them with all the options, and they had to choose between the two robots in Experiment I and the three robots in Experiment II, given their names and their position in the frame.
2) Specify whether the robot they chose was the one they watched tell a story at the beginning, with us giving them three options (Yes, No, and Do Not Remember).
3) With regard to the most important factor that led them to the robot of their choice, provide one out of three possible answers: a) the content of the robot’s story, b) the manner in which the robot told the story, and c) the robot’s movements. Participants could also provide their own answer. Finally, participants were asked their gender and age for demographical purposes.

#### Data Analysis

We used descriptive statistics to evaluate the proportion of participants who preferred each robot after witnessing the robots’ conversation and the reason why they chose it. We calculated the percentage of the participants who preferred a robot or an option given by the final choice questionnaire per condition.

In Experiment I, comparing two different robot conditions, we analyzed the questions of the KAT with the Mann–Whitney U test by using the sum of each participant per questionnaire. Similarly, for the JQ scores, we calculated a single number for each participant and applied the Mann–Whitney U test. To process KAT and JQ scores in Experiment II, we applied the Tukey post hoc test multiple comparisons to find any significant differences between the three groups and reduce the probability of family error. Those analyses were used to evaluate H3 and H4.

In both experiments, to highlight H1, a logistic regression analysis was performed to access the ability of a series of predictor variables, such as the individual robot storytelling that participants watched at the beginning of each experiment and the enjoyment level based on JQ and knowledge acquisition based on the KAT score to predict the participants’ preference after observing the robots’ conversation (López et al., 2015). If the robot that participants originally saw was proven to be a predictive factor for their choice after the conversation, we will accept H1. If not, we will decline H1. In case that the robot that participants originally saw was not proven to be a predictive factor and at the same time, based on the descriptive statistics, the most preferable robot was the cheerful one, we will accept H2. Additionally, we applied the logistic regression analysis to test H5. Moreover, to identify any statistically significant relationships between the variables, we applied an ANOVA analysis. López et al. recommended the use of a size effect analysis to indicate the power of the study’s sample (López et al., 2015) for gender unbalanced studies. Thus, we applied Hedges’ g analysis.

To statistically evaluate the proportion of participants who watch one robot’s storytelling individually and then choose the same robot (or another) after witnessing the robots’ conversation, we applied the McNemar test. The test is used to determine if there are differences in nominal dependent variables between two related groups. For the corresponding analysis in Experiment II, where there are three conditions, we applied the Cochran’s Q test. Moreover, we applied chi-square analysis to determine the number of participants who failed to recognize the robot that they originally watched in the individual robot’s storytelling after they were asked to during the preference questionnaire. More specifically, after the robots’ conversation, they were asked to choose with which robot they want to continue the storytelling task. After choosing one, they were asked if they chose the same robot as the one they had the storytelling from in the first place. Some of them thought that they chose the same one, although they did not, and we are interested in examining it. Table 1 shows the statistical analysis that has been used to support or decline each hypothesis.

**TABLE 1:** Statistical analysis that was applied to support or decline each hypothesis.

Hypothesis	Statistical analysis
H1	Logistic regression: if the robot that participants originally saw was proven to be a predictive factor for their choice after the conversation, we will accept H1. If not, we will decline H1.
	To statistically evaluate the proportion of participants who watch one robot’s storytelling individually and then choose the same robot (or another) after witnessing the robots’ conversation, we applied the McNemar test for experiment I and Cochran’s Q test for experiment II.
H2	Descriptive statistics
	Logistic regression: in case that the robot that participants originally saw was not proven to be a predictive factor and at the same time, based on the descriptive statistics, the most preferable robot was the cheerful one, we will accept H2
H3	Experiment I: Mann–Whitney *U* test; experiment II: Tukey *post hoc* test
H4	Experiment I: Mann–Whitney *U* test; experiment II: Tukey *post hoc* test
H5	ANOVA analysis: statistically significant relationships between the variables
	Logistic regression analysis for each condition by using the enjoyment level based on JQ and knowledge acquisition based on the KAT score to predict the participants’ preference after observing the robots’ conversation

### Experiment I: Serious vs. Cheerful-Personality Robot

In Experiment I, we introduced two different personality robots, namely, a serious and a cheerful one, in an individual interactive storytelling about different robots to assess the participants’ knowledge acquisition and level of enjoyment. After witnessing a conversation between the two robots, we asked the participants to choose one of them to collaborate with in the future. The purpose of Experiment I is the testing of H1, namely, whether the students will choose the robot that performed the storytelling in the first part of the experiment. If this hypothesis is confuted and the participants choose a different robot, based on H2, we expect that the preference would be the cheerful-personality robot. Similarly, we expect that participants experiencing the cheerful robot condition will have larger scores in the KAT (H3) and in the JQ score (H4).

#### Participants

The total number of participants was 107, aged 19–48 years. There were 57 of them in the serious robot condition (Alfa): 47 women (82.5%), 7 men (12.3%), and 3 (5.3%) who prefer not to mention their gender. The 50 participants in the cheerful robot condition (Omega) were 45 women (90%), 4 men (8%), and 1 who prefers not to mention their gender, aged 19–48 years. In the control condition, where participants watch only the robots’ conversation, were 25 people, aged 20–54 years—22 women (88%) and three men (12%).

#### Storytelling Example

The text quoted is a translation from the original Greek text.

Cheerful Robot (Omega): “*Hello and welcome to our exhibition on robots and their history. I hope you like robots, not only because I am a robot too but because since you are here, you will hear the story of eight robots who are my friends. But even if you do not like them, I suggest pretending that you do so as not to disappoint me. I am very happy to talk to you about them!... So let’s start with the athlete of our team, TOPIO, or as his acronym means, a robot that plays ping pong. It is a humanoid robot designed to play table tennis against a human. And believe me, it’s very good at it* …”

Serious Robot (Alfa): “*Good evening and welcome to our exhibition on robots and their history. I will be your guide and I am going to talk to you about eight robots … Let’s start with TOPIO, or as its acronym translates to, a robot that plays ping-pong. It is a humanoid robot designed to play ping-pong against a human* …”

Conversation Example:

‘*A: Good evening my dear colleague, how did the activity with the students go?*

O: I am very happy, and I am always very happy to talk to them. How about you?

A- I also always appreciate our interaction and I feel lucky that I can pass on knowledge to them.

O- Isn’t it amazing that today we talked to them about our friends?

A-every time I admire your enthusiasm when you talk about a topic.

*O- I admire your professionalism every time*.’

#### Procedure

The procedure is the same as that described in *General Method*. At the beginning, participants witnessed one of the robots (cheerful or serious) individually doing the storytelling (Part 1) and, subsequently, the conversation between those two robots (Part 2).

#### Results

The Hedges’ g value was calculated to be 6.506, which represents an acceptable “medium” effect size. There were no statistically significant differences between the participants’ KAT scores in the cheerful robot condition (*M* = 15.62, *SD* = 0.22) and the serious robot condition (*M* = 17.57, *SD* = 0.41), as indicated by the U-test (U = 123, *p* = 0.197, r = 1.29). Similarly, there were no statistically significant differences in the JQ scores between the cheerful (*M* = 131.04, *SD* = 0.87) and the serious robot conditions (*M* = 134.95, *SD* = 0.54), as indicated by the U-test (U = 124, *p* = 0.26, r = 1.12). Moreover, the individual robot that participants watched in the first part of the experiment (cheerful or serious) was not associated with greater odds of choosing it again after the robots’ conversation (beta = −0.004, stdError = 0.114, t = −0.036, *p* = 0.972). Similarly, no effect was found with the KAT scores (beta = −0.144, stdError = 0.007, t = −1.476, *p* = 0.143) and with the JQ scores, as indicated by the logistic regression analysis (beta = −0.091, stdError = 0.002, t = −0.912, *p* = 0.364). Results were also confirmed by the corresponding ANOVA analysis (F_(2, 52)_ = 1,066, *p* = 0.367).

The participants’ preference after witnessing the robots’ conversation was biased toward the cheerful robot. As indicated by the McNemar test, participants statistically significantly preferred the cheerful robot after witnessing the robots’ conversation (*p* = < 0.001). A total number of 79 participants preferred the cheerful robot. The 75.4% (N = 43) of those 79 who preferred the cheerful robot came from the group who originally watched the serious robot performing the individual storytelling, and 72% (N = 36) of them came from the group who witnessed the cheerful robot. The serious robot was chosen by a total of 28 participants, 14 from each of the two conditions (serious and cheerful).

Based on the McNemar test, the proportion of participants who watch the cheerful robot individually and choose it after the conversation (36/50 = .72^2^) is similar to that of those who watch the serious robot performing the individual storytelling but choose the cheerful robot after witnessing the conversation (43/57 = .75^3^).

The participants’ self-reported answers about whether they chose the same robot as the one who originally performed the individual storytelling are detailed in Table 2, which also lists the reasons for their choice. Eight participants specified their own reasons in addition to those that were listed to explain their choice. Some of the participants reported that they chose the same robot as the one that they originally saw; however, this was not true. The number of participants who failed to recognize the robot that they originally saw (in both serious and cheerful robot conditions) was not statistically significant in comparison with that of those who perceived it correctly, as indicated by X^2^ (1, N = 107 = 0.315, *p* > 0.575).

**TABLE 2:** Experiment I. Total number and percentages of participants’ answers in preference questions.

Did you choose the same robot ?	Preference questions		
	Yes	No	Don’t remember
Serious robot	35.1% (N = 20)	49.1% (N = 28)	15.8% (N = 9)
Cheerful robot	56% (N = 28)	28% (N = 14)	16% (N = 8)

Why?	Storytelling content	Way (both storytelling and movements)	Movements
Serious robot	10.5% (N = 6)	64.9% (N = 37)	15.8% (N = 9)
Cheerful robot	24% (N = 12)	60% (N = 30)	10% (N = 5)

Finally, those who participated in the control condition and saw only the robots’ conversation preferred the cheerful robot in accordance with the rest of the participants at 80%.

#### Discussion

The clear winner in terms of participants’ preference was the cheerful robot, confirming H2. H1 was not confirmed, as there was no relationship between the first robot in the storytelling sequence and the participants’ choice after the robots’ conversation, based on the reported nonsignificant logistic regression coefficients. Similarly, participants from the control condition, who did not experience the storytelling task and did not see each robot separately, had the same preference as other participants. The fact that participants exhibited similar knowledge acquisition, as indicated by KAT scores and the level of enjoyment indicated by JQ scores, shows that both robots are suitable for the proposed activity. Furthermore, we noted the difficulty of the participants to realize which robot they had originally observed, although the effect is not statistically significant. Finally, the absence of coefficient correlation between the participants’ KAT and JQ scores was expected, since in both conditions, participants had similar results.

### Experiment II: Expressive Movement vs. Friendly Storytelling

In Experiment II, we highlighted different robot modalities based on the findings in Experiment I. We deconstructed the modalities of the cheerful robot (the preferred robot) to be expressive body movements and friendly storytelling. Based on these two characteristics, we designed two cheerful robot characters, one with intense expressive body movements (Hta) and one with extremely friendly storytelling (Kapa). The extreme variation of the conditions was based on psychological testing methodology (Urbina, 2014). The cheerful robot exhibits baseline behavior, performing both expressive body movements and friendly storytelling, similarly to Experiment I. Hta maintains the exact same script with additional body movements. Furthermore, Kapa is enhanced with friendly phrases in storytelling and less expressive body movements. The purpose of Experiment II was to test H1 and investigate H5.

#### Participants

The total number of participants in experimental conditions was 177, 85.87% women, 8.47% men, and 5.64% who preferred not to mention their gender. In the cheerful robot condition participated 63 students, 59 in intense expressive movements and 55 in friendly storytelling, aged between 18 and 52 years with similar characteristics to those in Experiment I, and correspondingly, the control group consisted of 25 participants, aged 26–53 years.

#### Storytelling Example

The three robots followed the cheerful robot storytelling from Experiment I. The cheerful robot (O) storytelling is identical to the one mentioned in 2.2.2, since it is exactly the same robot, while the script in the intense expressive body movements (H) is also identical, enhanced with extremely expressive body movements, as shown in the accompanying video. Finally, for the friendly storytelling robot (K), the script is enhanced with friendly phrases that are commonly used in the Greek language, such as addressing the participants as “friends” and using the phrase “my friend” between the sentences. An example follows:

‘*Hello my friends and welcome to our exhibition on robots and their history … But even if you do not like them my friends, I suggest pretending that you do so as not to disappoint me…. So my friends, let’s start with the athlete of our team, TOPIO,*’

Conversation:

‘…*O- Did you manage to talk to them (participants) about all the robots? I’m so glad to have the opportunity to explain, that robots are not just those that look like us, but also machines that look like airplanes*.

*H: Yes, I made it to talk to them about both humanoids and unmanned aircrafts. It is important for them to understand that robots are not called robots only based on their appearance but also by their intelligence and mechanical characteristics.*

*K- But of course our friends would understand, they all seemed happy and friendly and I felt that they were having fun with what I was telling them.*

*O: They also seemed excited and thirsty for learning.*

*H- Based on our discussion, it seems that we have a different point of view about the ideal storytelling style.*

*K- Indeed my friends. I prefer to cultivate a friendly relationship and talk to them as if we have known each other for years. I express my opinion, and I will slip my tongue and say phrases like ‘oh my friend’ or ‘friends’.*

*O- I, on the other hand, am not addressing them personally and when something makes me happy, I show it to them, tell jokes and express my opinion.*

*M- I, from my side, accompany what I say with strong movements. What would you say before we return to the activity to show in practice our storytelling techniques?..’*

#### Procedure

The procedure was the same as that described in *General Method*. At the beginning, participants witnessed one of the three robots (Omega, Hta, or Kapa) doing the storytelling individually (Part 1) and, subsequently, a conversation between those three robots (Part 2), as shown in Figure 3, regarding their storytelling techniques, as reported in 2.3.2.

#### Results

The total average KAT score, which represents the participants’ knowledge acquisition in the cheerful robot condition, was close in all robot conditions. In closer detail, there is no statistically significant difference between the cheerful robot condition (*M* = 16, *SD* = 1.63) and the expressive movements condition (*M* = 14.91, *SD* = 1.92), as indicated by the Tukey HSD test at *p* = 0.63 and the friendly storytelling (*M* = 14.04, *SD* = 1.46) at *p* = 0.98. Similarly, nonsignificance is also indicated by the relationship between the expressive movements and friendly storytelling robots at *p* = 0.642. The total average JQ scores were also similar between the cheerful robot condition (*M* = 142.11, *SD* = 2.34) and the expressive movements robot condition (*M* = 135.93, *SD* = 2.36), as indicated by the multiple comparison test at *p* = 0.97 and friendly storytelling (*M* = 142.51, *SD* = 2.5) at *p* = 0.325. The relationship between the expressive movements robot condition and the friendly storytelling robot condition were also comparable, at *p* = 0.331.

The ANOVA results indicated that there is a dependence relationship between the variables (F_(2, 55)_ = 4.29, *p* = 0.008). Moreover, based on the logistic regression analysis, the robot that participants originally saw in the first part of the experiment was associated with greater odds of choosing it again after the robots’ conversation (beta = 0.330, stdError = 0.082, t = 1.33, *p* = 0.05). A similar effect was found in the relationship between participants’ choice and their JQ scores (beta = 0.128, stdError = 0.003, t = −0.419, *p* = 0.004). In other words, participants who evaluated the individual robot’s storytelling with higher JQ scores had greater odds to choose it again after witnessing the robots’ conversation. On the contrary, KAT scores were not found to be predicted factors for participants’ preference (beta = −0.032, stdError = 0.01, t = −0.419, *p* = 0.679).

A total of 82 participants preferred the cheerful robot, 65 participants the expressive movements, and 30 the friendly storytelling robot. From those who evaluated the robots after originally having a storytelling experience with the Kapa robot (friendly storytelling), 38.2% (N = 21) preferred the expressive movements robot, 50.9% (N = 28) the cheerful one, and the remaining six of them the Kapa.

From those who originally watched the individual storytelling of the cheerful robot, 50.9% (N = 32) preferred the cheerful robot, 31.7% (N = 20) the expressive movements, and 17.5% (N = 11) the friendly storytelling.

The participants’ preference after witnessing the robots’ conversation is not statistically significant between the three conditions, as indicated by the Cochran’s Q test, at *p* = 0.087.

**FIGURE 4:**
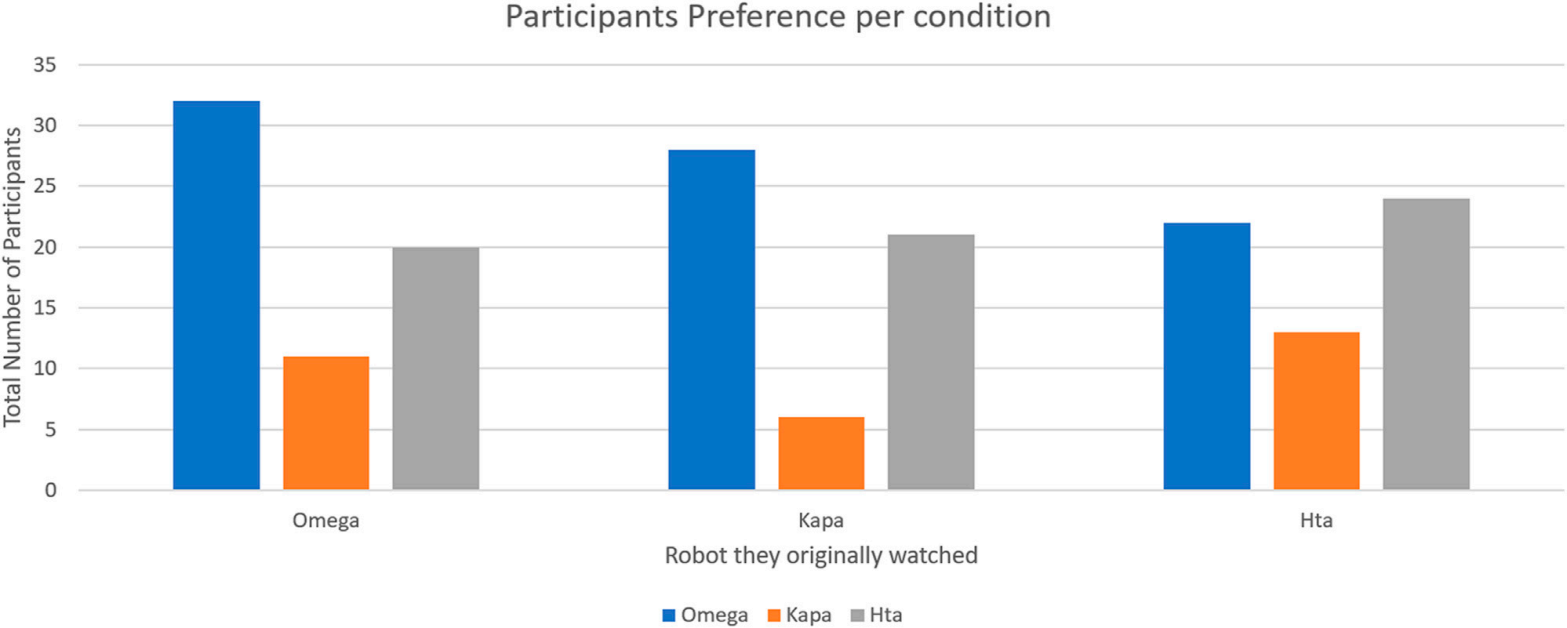
Participants’ preferred robot, from each condition (color).

Finally, from those who participated in the expressive movements robot’s condition, 40.7% (N = 24) preferred the same robot, 37.3% (N = 22) the cheerful robot, and 22% (N = 13) the friendly storytelling robot. The participants’ preferences per condition are graphically presented in Figure 4.

Those who participated in the control condition here witnessed only the robots’ conversation and preferred the cheerful robot to perform an educational museum storytelling at 70.6%. On the other hand, 23.5% preferred the intense body movements one and 5.9% the friendly storytelling one.

Participants’ self-reported answers if they preferred the original robot after the robots’ conversation and the most representative reason behind their preference are listed in Table 3. Similar to Experiment I, a few participants failed to recognize the robot they originally saw, thinking that after the robots’ conversation, they preferred the robot they originally saw. The number of participants who failed to recognize the robot they preferred from the expressive movement condition (N = 6) was similar to that of those who participated in the extremely friendly condition (N = 4), as indicated by X^2^ (1, N = 60 = 0.121, *p* = 0.728). As for those who participated in the cheerful robot condition, only two participants failed to correctly recognize the robot they originally watched when they preferred it after the conversation.

**TABLE 3:** Participants’ preference question results.

Did you choose the same robot ?	Preference questions		
	Yes	No	Don’t remember
Cheerful robot	46% (N = 29)	34.9% (N = 22)	19% (N = 12)
Expressive movements robot	37.3% (N = 22)	45.8% (N = 27)	16.9% (N = 10)
Friendly robot	43.6% (N = 24)	41.8% (N = 23)	14.5% (N = 8)

Why?	Storytelling content	Way (both storytelling and movements)	Movements
Cheerful robot	15.9% (N = 10)	60.3% (N = 38)	19% (N = 12)
Expressive movements robot	13.6% (N = 8)	47.4% (N = 18)	42.1% (N = 16)
Friendly robot	12.7% (N = 7)	52.5% (N = 31)	30.5% (N = 18)

#### Discussion

Results show that the level of enjoyment from the original robot’s storytelling leads to higher odds of being chosen by the participants after the robots’ conversation. Importantly, those who participated in the control condition had a different preference than those who observed the storytelling task and the robots individually. Our result show that knowing the task and seeing the robot in action affects their preference, as was also indicated by Velentza et al. (2020), where future teachers evaluated different robot characteristics as more important before and after seeing the Nao robot teaching in a university classroom. Furthermore, in the cheerful and expressional movement robot condition, most participants preferred the robot that originally did the storytelling. The extremely friendly storytelling robot was deemed as the least appropriate for performing the task, as it was by far the less preferable. However, we found no statistically significant differences between the KAT and JQ scores among the conditions.

## General Discussion

Our study focuses on identifying robot characteristics that enhance the audience’s knowledge acquisition and level of enjoyment from the performed activity and, most importantly, that make people want to collaborate with the robot again for future activities. We were mainly interested in the robot’s personality and different modalities. Thus, we compared a serious- and a cheerful-personality robot regarding the terms of the participants’ gained knowledge scores from the corresponding multiple-choice questionnaire (KAT) and the level-of-enjoyment score based on the self-reported Likert scale questionnaire (JQ). After participants experienced storytelling by one of the robots for 10 min and filled in the questionnaires, they watched a conversation between the robots and chose one of them to continue the storytelling activity. Results demonstrate that both cheerful and serious robots are appropriate for the task, based on the participants’ knowledge acquisition and enjoyment level scores. Nevertheless, the participants preferred the cheerful robot, confirming H2. The same result was obtained from the control group, where participants had no prior experience with the robot performing the task of storytelling. Apart from the participants’ performance assessment, based on Robert’s findings (Robert, 2018), we investigated whether they would choose to continue the activity with the robot they originally observed (H1). In Experiment I, H1 was not confirmed since most of the participants from all conditions preferred the cheerful one.

Following the participants’ lead that a cheerful-personality robot is preferable, we pinpointed the modalities that give it its cheerful character to be expressive body movements and friendly storytelling. Numerous researchers have shown the importance of both those modalities in the robot’s emotional expression (Striepe et al., 2019; Salem et al., 2012; Aly and Tapus, 2016). Thus, we investigated whether any modalities are more decisive in the human–robot interaction. This finding is crucial for robot designers, constructors, and researchers.

To the best of our knowledge, this study is the first to investigate robot personality traits and modalities simultaneously. Furthermore, it provides participants with the opportunity to witness conversation between robots with up to three different modalities. Our findings are in line with those from Mikata et al., 2019, stating that a combination of modalities is important for human–robot interaction. Furthermore, expressive body movements are considered as the most important factor for the participants’ choice. This was confirmed by the participants’ answers when asked to support the reasons for their preference. Most participants (69.3%) claimed that the robot’s manners, a combination of storytelling and movements, was important; second, they stated that the robot’s movements made them choose it. However, our results clearly demonstrate that a robot performing extremely friendly storytelling is not preferable to the participants for educational activities.

The participants’ prior experiences with a robot integrated with functional behaviors can influence their preference after witnessing the robots’ conversation. Logistic regression analysis indicated that participants were more likely to choose the original robot after the robots’ conversation if they had high JQ scores and participated in the cheerful or the expressive movement condition. We assume that the participants’ preference seems to be a two-stage process. The two robots were first evaluated as more appropriate for the task. Then, after participants “checked” in their minds that those two are more suitable, they chose the one they originally had the storytelling with, partially confirming H1. First, a general evaluation is conducted, and afterward, if the choice is between two robots that are both appropriate for the task, participants turn toward the one they are familiar with. Knowledge of the task also plays a crucial role in the participants’ preference, as previously indicated by Velentza et al. (2020). In Experiment II, participants in the control group, without having any experience with the task, significantly preferred the cheerful robot, while those who experienced the storytelling task from the first part of the experiment, at a high percentage, preferred the expressively moving robot.

In future work, it will be interesting to apply a correlation between the frequency of the cheerful terms and phrases in the robot’s storytelling and the response of the participants and perhaps even a peak-shift effect. We also encourage other research groups to manipulate the frequency of cheerful phrases in robot storytelling. There are also some limitations that need to be discussed. The task was a specific educational activity in a museum environment. The results cannot be generalized in different environments without further testing. Additionally, the storytelling was about the history of robots. Although in similar experimental designs with different storytelling, that is, modern art paintings in the studies by Velentza et al., 2019, Velentza et al., 2020, participants had a higher level of enjoyment with the cheerful personality robot, we cannot exclude the possibility that the story would influence the participants to prefer a robot. Another limitation of the study is the robots’ embodiment, which was *via* video annotation and not with a physical presence. Although there have been similar studies in the past which successfully evaluated robots’ behavior *via* video (Walters et al., 2011; Li et al., 2016), there are also others who found differences in participants’ learning outcome after comparing virtual robots with present robots (Cruz-Maya and Tapus, 2016). Thus, in order to make sure that our results can be generalized outside the video presence of robots in a real-world environment, as future work, we are planning to replicate the experiment in real-environment conditions with robots making a physical appearance in front of the participants. Moreover, in terms of result generalization, we encourage researchers to replicate the experiment in different populations, other than college students, and we are also planning to replicate it in an actual museum environment. Most of the students in educational schools are women, and thus, the experiment’s replication in different populations will give interesting results regarding potential gender differences between participants’ preferences.

Overall, we propose the use of cheerful personality robots with expressive body movements and cheerful storytelling, which do not cross the line between being friendly and extremely friendly. Moreover, we believe that our results can also be applied beyond the specific activity of storytelling.

## Data Availability

The datasets presented in this study can be found in online repositories. The names of the repository/repositories and accession number(s) can be found in the article/Supplementary Material.

## Footnotes

1
https://filmora.wondershare.com

2 Number of participants who originally saw the robot and choose it after the conversation/total number of participants who participated in the robot’s condition.

3 Total number of participants who did not see the robot, although they choose it after the conversation/sum of participants who did not see it but choose it and did not see it and did not choose it either.
