# Supplementary material for: Which One? Choosing Favorite Robot After Different Styles of Storytelling and Robots’ Conversation
Source: Front Robot AI. 2021 Sep 9;8:700005. doi: 10.3389/frobt.2021.700005 (PMC8458710; doi:10.3389/frobt.2021.700005)
Supplement: Supplementary file 2 [file DataSheet1.docx]

# Data Set

## Experiment I

Table 1: *Comparison between the learning outcome (KAT Scores) of participants who originally witnessed the cheerful robot performing the storytelling vs those who witnessed the serious one.*

|  | (I) Condition I | (J) Condition J | Mean Difference (I-J) | U | Sig. |
| --- | --- | --- | --- | --- | --- |
|  |  |  |  |  |  |
| Mann Witney U Test | Serious | Cheerful | 1.29 | 123 | .197 |

Table 2: *Comparison between the level of enjoyment (JQ Scores) of participants who originally witnessed the cheerful robot performing the storytelling vs those who witnessed the serious one.*

|  | (I) Condition I | (J) Condition J | Mean Difference (I-J) | U | Sig. |
| --- | --- | --- | --- | --- | --- |
|  |  |  |  |  |  |
| Mann Witney U Test | Serious | Cheerful | 1.12 | 124 | .26 |

Table 3: *Logistic regression analysis to access the ability of a series of predictor variables, between participants who watched the cheerful robot and participants watched the serious robot at the beginning of each experiment, enjoyment level based on JQ and knowledge acquisition and based on the KAT score to predict the participants’ preference after observing the robots’ conversation.*

| **ANOVA^b^** | | | | | | |
| --- | --- | --- | --- | --- | --- | --- |
| Model | | Sum of Squares | df | Mean Square | F | Sig. |
| 1 | Regression | ,802 | 3 | ,267 | 1,066 | ,367^a^ |
|  | Residual | 25,834 | 103 | ,251 |  |  |
|  | Total | 26,636 | 106 |  |  |  |
| a. Predictors: (Constant), enjoy_level, score, robot_choice | | | | | | |
| b. Dependent Variable: robot_video | | | | | | |

| **Coefficients^a^** | | | | | | |
| --- | --- | --- | --- | --- | --- | --- |
| Model | | Unstandardized Coefficients | | Standardized Coefficients | t | Sig. |
|  |  | B | Std. Error | Beta |  |  |
| 1 | (Constant) | 1,953 | ,359 |  | 5,445 | ,000 |
|  | robot_choice | -,004 | ,114 | -,004 | -,036 | ,972 |
|  | Kat_score | -,011 | ,007 | -,144 | -1,476 | ,143 |
|  | enjoy_level | -,002 | ,002 | -,091 | -,912 | ,364 |
| a. Dependent Variable: robot_video | | | | | | |

Experiment II

Table 4: *Comparison between the learning outcome scores (KAT) of participants who watched the cheerful robot (O), the extremely body movements robot (H) and friendly storytelling robot (K)*

| **Multiple Comparisons** | | | | | | | |
| --- | --- | --- | --- | --- | --- | --- | --- |
| Dependent Variable: LEARNING (KAT) | | | | | | | |
|  | (I) VAR. NAME | (J) VARIABLES | Mean Difference (I-J) | Std. Error | Sig. | 95% Confidence Interval | |
|  |  |  |  |  |  | Lower Bound | Upper Bound |
| Tukey HSD | Exp. Movement (H) | K | 1.2173 | 1.50263 | .642 | -2.3498 | 4.7844 |
|  |  | O | -.8637 | 1.37998 | .631 | -4.1397 | 2.4122 |
|  | Friendly Storytelling (K) | H | -1.2173 | 1.50263 | .642 | -4.7844 | 2.3498 |
|  |  | O | -2.0810 | 1.46576 | .980 | -5.5606 | 1.3986 |
|  | Cheerful (O) | H | .8637 | 1.37998 | .631 | -2.4122 | 4.1397 |
|  |  | K | 2.0810 | 1.46576 | .980 | -1.3986 | 5.5606 |
| Based on observed means.  The error term is Mean Square(Error) = 41.283. | | | | | | | |

Table 5: *Comparison between the level of enjoyment scores (JQ) of participants who watched the cheerful robot (O), the extremely body movements robot (H) and friendly storytelling robot (K)*

| **Multiple Comparisons** | | | | | | | |
| --- | --- | --- | --- | --- | --- | --- | --- |
| Dependent Variable: Level of Enjoyment (JQ) | | | | | | | |
|  | (I) VAR. NAME | (J) VAR00002 | Mean Difference (I-J) | Std. Error | Sig. | 95% Confidence Interval | |
|  |  |  |  |  |  | Lower Bound | Upper Bound |
| Tukey HSD | H |  |  |  |  |  |  |
|  |  | K | -8.8256 | 6.80123 | .331 | -26.5434 | 8.8923 |
|  |  | O | -1.9152 | 6.24610 | .972 | -18.1868 | 14.3565 |
|  | K |  |  |  |  |  |  |
|  |  | H | 8.8256 | 6.80123 | .331 | -8.8923 | 26.5434 |
|  |  | O | 6.9104 | 6.63434 | .325 | -10.3727 | 24.1935 |
|  | O |  |  |  |  |  |  |
|  |  | H | 1.9152 | 6.24610 | .972 | -14.3565 | 18.1868 |
|  |  | K | -6.9104 | 6.63434 | .325 | -24.1935 | 10.3727 |
| Based on observed means.  The error term is Mean Square(Error) = 845.747. | | | | | | | |

Table 6: *Logistic regression analysis to access the ability of a series of predictor variables, between participants who watched the cheerful robot (O), the extremely body movements robot (H) and friendly storytelling robot (K) at the beginning of each experiment, enjoyment level based on JQ and knowledge acquisition and based on the KAT score to predict the participants’ preference after observing the robots’ conversation.*

| **Coefficients^a^** | | | | | | |
| --- | --- | --- | --- | --- | --- | --- |
| Model | | Unstandardized Coefficients | | Standardized Coefficients | t | Sig. |
|  |  | B | Std. Error | Beta |  |  |
| 1 | (Constant) | 2,185 | ,540 |  | 4,049 | ,000 |
|  | Robot Choice | ,202 | ,330 | ,330 | 1.31 | ,050 |
|  | JQ | -,127 | ,003 | ,128 | -4,19 | ,004 |
|  | KAT | ,090 | ,010 | -,032 | -,419 | ,679 |
| a. Dependent Variable: Repeat Choice | | | | | | |
